# Internet-Based Cognitive Behavioral Therapy Among Psychologists in a Medical Setting: A Survey on Implementation

**DOI:** 10.2196/13432

**Published:** 2019-08-09

**Authors:** Renée V H IJzerman, Rosalie van der Vaart, Andrea W M Evers

**Affiliations:** 1 Unit of Health, Medical and Neuropsychology Faculty of Social and Behavioural Sciences Leiden University Leiden Netherlands

**Keywords:** eHealth, chronic care, self-management, implementation, psychologists, UTAUT

## Abstract

**Background:**

Internet-based cognitive behavioral therapy (iCBT) is an effective treatment for patients with a chronic somatic illness to improve self-management skills and to learn to adjust to their chronic disease and its impact on daily life. However, the implementation of iCBT in clinical practice is challenging.

**Objective:**

This study aimed to examine the current degree of implementation of iCBT among psychologists in a medical setting and discover determinants influencing the implementation of iCBT among nonusers.

**Methods:**

A Web-based survey, based on the Unified Theory of Acceptance and Use of Technology (UTAUT), was distributed among psychologists in a medical setting. The survey included questions regarding the current use of iCBT, intention to use iCBT in the future, and operationalized concepts of the UTAUT constructs, that is, performance expectancy (PE), effort expectancy (EE), social influence (SI), and facilitating conditions (FC).

**Results:**

In total, 107 (24.8%) psychologists completed the survey. Of them, 16.8% have access to iCBT, 15.9% currently use iCBT, and 21.5% are expected to use iCBT within the next year. The constructs PE, EE, and SI together significantly influenced behavioral intention (BI; mean 3.9 [SD=0.8]) among nonusers (*R*^2^=0.490; *F*_4.85_=20.405; *P*<.001).

**Conclusions:**

In spite of an average to high BI, the current implementation of iCBT is rather low among psychologists in a medical setting. Further research should focus on reducing the gap between intention to use and actual use by focusing on influencing the predictive UTAUT constructs.

## Introduction

### Background

Patients with a chronic somatic illness often struggle with a complex interaction of physical, psychological, and social demands related to their disease. These demands significantly influence their disease perception and quality of life [1-3]. Supporting patients in increasing their disease self-management can effectively increase illness adjustment and adherence to treatment, and decrease problems related to illness behaviors and comorbid mental illnesses such as depression and anxiety [4-6]. Psychologists can offer this support as part of outpatient treatment in a hospital clinic. In the Netherlands, these psychologists are called medical psychologists, and patients are referred to them by their medical specialist. Regarding the content of this support, cognitive behavioral therapy (CBT)–based techniques are regularly used. However, CBT is generally only available to a small number of patients in this setting because it is expensive, time consuming [7,8], and sometimes inaccessible because of attendance barriers [9,10].

### Internet-Based Cognitive Behavioral Therapy

With the development of internet-based treatment [11], CBT has become much more accessible to a wide range of patients [4]. In the Netherlands, internet-based CBT (iCBT) is usually offered as a guided program, containing modules regarding goal setting, psychoeducation, (behavioral) assignments, relaxation exercises, and diary registrations, which are supported by asynchronous contact with a therapist through messages in a secured email box [12]. The iCBT service can be used in several ways, depending on the severity of the problems of the patient and the patient preferences. It can be provided as a stand-alone service, in which it is a replacement of a regular therapy, or it can be used as an addition to regular treatment, in which iCBT is used blended, combined with face-to-face sessions. In all scenarios, patients use the Web portal from their home environment. Use of iCBT potentially produces similar overall effects compared with face-to-face CBT [13-15] and is significantly efficacious in improving disease-specific symptoms, disease control [9,16-18], and disease-related physical outcomes [13]. Moreover, treatment by traditional forms of psychological therapy can be associated with stigmatizing beliefs about mental illness and negative prejudices about therapists [19], whereas iCBT is usually experienced as easy to access and the most private way to seek help [20]. Moreover, iCBT provides patients the ability to administer treatment and access treatment-related material at any time and any place [16]. Finally, regarding economical perspectives, offering iCBT enables a potential reduction in health care costs [21], the number of needed contact hours, waiting lists, and traveling time for patients [22].

### Current Implementation of Internet-Based Cognitive Behavioral Therapy

However, implementation of iCBT in clinical practice remains a challenge [23,24], and different health care settings show different usages of iCBT. Results of a naturalistic study among therapists of a Dutch mental health center showed that after training, over a period of 3 years, only 3.6% of the patients were offered the possibility to participate in iCBT, initiated by 18% of the therapists qualified to provide iCBT [25]. On the contrary, a study among Dutch primary care psychologists (PCPs) and mental health counselors (MHCs) in general practitioner practices showed more optimistic numbers, with 29% of the PCPs and 60% of the MHCs having used Web-based psychological self-management interventions (based on CBT) in their treatments [26]. Regarding iCBT in the hospital setting, research is lacking. Therefore, the aim of this study was to gain insight into use, barriers, and facilitators regarding implementation of iCBT among psychologists in medical settings.

Research into possible factors influencing the degree of implementation of iCBT and iCBT-related interventions among psychologists is relatively young. Dissemination and implementation of digital health interventions often face many barriers on differing levels, for example, related to perceived effectiveness, expected usability, usefulness in the patient population, facilitating conditions (FC) at work, and personal productivity [26,27]. A suitable model to measure acceptance of technology and its associated influences on those multiple levels is the Unified Theory of Acceptance and Use of Technology (UTAUT) [28]. On the basis of this model, determinants influencing behavioral intention (BI) of iCBT are mapped, related to future users’ performance expectancy (PE), effort expectancy (EE), social influence (SI), and available FC. [28].

### Use of the Unified Theory of Acceptance and Use of Technology

According to the UTAUT model, 4 constructs are crucial in explaining the use of technology, namely *PE, EE*, *SI*, and *FC*. *PE* indicates “the degree to which an individual believes that using the system will help him or her to attain gains in job performance” [28]. For psychologists in medical settings, this would mean that the use of iCBT may or may not contribute to their quality of work in a positive manner, for instance, the use of iCBT reduces time that is required to fulfill major responsibilities (eg, providing help to patients). *EE* can be defined as “the degree of ease associated with the use of the system” [28]. In practice, this would mean that the use of iCBT may or may not be clear, understandable, and easy to learn for psychologists. *SI* indicates “the degree to which an individual perceives that important others believe he or she should use the new system” [28]. In practice, this would mean that psychologists may or may not believe that colleagues and management at the workplace think they should use iCBT and support their use of iCBT. *FC* indicates “the degree to which an individual believes that an organizational and technical infrastructure exists to support the use of the system” [28]. For psychologists in medical settings, this would indicate that the use of iCBT may or may not be stimulated by the organization or manager. Moreover, the construct *BI* is a crucial determinant for use behavior and can be defined as “a person's subjective probability to perform a specified behavior” [29]. In practice, this would mean the likelihood of use of iCBT among psychologists in medical settings.

The purpose of this study was to examine the fit and current degree of implementation of iCBT among psychologists in a medical setting and explore the extent to which the determinants of the UTAUT model influence the degree of implementation among nonusers. This was done using a nationally spread survey in the Netherlands to gain broad and generalizable insights into barriers and facilitators regarding implementation of iCBT in hospitals.

## Methods

### Recruitment

Respondents were recruited by (personal) email in March 2017. Dissemination of the survey was done by the Dutch Association of Medical Psychologists, and email addresses were collected by systematically searching hospitals per province (using Zorgkaart Nederland and Google), after which these hospitals were approached. In case no information on names or email address of psychologists was found on the website, the secretariat of the hospital was contacted by phone to ask whether they would agree to share their psychologists’ contact information. This resulted in a total initial spreading among 432 addresses. However, a snowball effect might have occurred because psychologists were asked to forward our invitation to colleagues to whom it might be relevant as well.

The email included an information letter and a link to the Web-based survey in Qualtrics (2015 Qualtrics, LLC). The information letter explained the purpose of the study and its voluntary nature, use and anonymization of the data, estimated time needed to participate (10 min), and the reward for participating in the study (5 gift certificates of €50 were raffled among the respondents). All email addresses were stored separately and were only used for the raffle. The Web-based survey started with an informed consent form. Not responding to the survey or opting out in the informed consent form was considered as choosing not to participate in the study. Psychologists who were employed at multiple hospitals were asked to choose 1 hospital and fill in the survey based on their chosen hospital. If the survey was not fully completed, data were not included in further statistical analysis. Furthermore, 1 and 2 weeks after sending the invitation emails, reminder emails were sent to all email addresses. The study was approved by the Psychology Research Ethics Committee of Leiden University.

### Survey

The survey consisted of 3 parts, namely (1) background information about the psychologist and the hospital, (2) general and work-related use of the internet of the respondent, and (3) an operationalization of the UTAUT constructs. The background information included gender, age, professional background, number of working hours per week, and years of employment. Background information about the hospital included number of colleagues working as a psychologist, number of newly referred patients per month, reasons for referral of patients, consult duration per visit, number of contacts per patient per treatment trajectory, and type of primarily offered help to patients. In part 2, general and work-related use of the internet was measured using questions about quantity of internet use in general, self-perceived skills to use the internet, use of internet for work-related activities, and current available Web-based applications in the hospital.

To operationalize the UTAUT, subscales were created for each construct in the model (PE, EE, SI, FC, and BI). Regarding BI, respondents first answered whether using iCBT was part of their current work activities, after which they had to answer whether they had the intention to start using iCBT in case they currently did not use it. By measuring actual use of iCBT, a distinction between users and nonusers was possible. Subscales of the UTAUT constructs were measured with 3 to 8 items formulated as statements, for instance, “I expect/experience that guided iCBT is effective for my patient population” (PE), “I expect/experience that guided iCBT is time intensive to use” (EE), “I expect/experience that iCBT is seen as a positive development among my colleagues” (SI), “I expect/experience that iCBT fits within the technological circumstances of my practice” (FC), and “I want to use/continue to use guided iCBT” (BI). Respondents answered statements on a 5-point Likert scale, ranging from completely disagree (1) to completely agree (5). A complete overview of the survey is provided in Multimedia Appendix 1.

### Statistical Analysis

Statistical analyses were performed using the Statistical Package for the Social Sciences (IBM SPSS Statistics 24). Descriptive statistics were used to describe the study sample, their internet experience, and their scores on the UTAUT constructs. Scale scores and Cronbach alphas were calculated for each UTAUT construct to check internal reliability. In the sample of nonusers, Pearson correlations were calculated to determine the relationship between the UTAUT variables PE, EE, SI, and FC and the dependent variable BI. On the basis of the statistical significance of these relations, multiple regression analysis was carried out to examine the further nature of these relations.

## Results

### Respondents

A total of 127 psychologists in medical settings filled in the survey. Among them, 107 completed the survey, whereas 20 stopped early. These data were not used for analyses and contained 11.8% of the data. Among the respondents, 58 (58/107, 54.2%) stated that they are members of the Dutch Association of Medical Psychologists, whereas 49 (49/107, 45.8%) stated that they are not members. The estimated response rate is 24.8%, as 432 people were contacted for participation via their email addresses. However, because of the method of data collection, it is not possible to calculate an exact response rate. Table 1 provides an overview of the respondents’ characteristics. Most respondents were female (92/107, 86.0%), with a mean age of 40.9 years (SD=11.3). A large part of the respondents had a professional background as a health care psychologist (45/107, 42.1%) or clinical psychologist (27/107, 25.2%; these are both protected titles in the Netherlands, based on postmaster educational tracks). On average, respondents have been active as psychologists in a medical setting for 10.0 (SD=8.1) years and received up to 20 (69/107, 64.5%) new patients each month (data not shown in Table 1). Regarding consultations, most patients were seen for 5 to 10 times (60/107, 56.1%), and therapy sessions lasted an average of 53 min per session. With regard to characteristics of the care provided, respondents most often reported treating problems with dealing with chronic physical complaints and limitations (93/107, 86.9%), anxiety- and/or mood-related problems (68/107, 63.6%), and pain (39/107, 36.4%). Finally, the type of care primarily provided consisted of interventions aimed at improving mental functioning (93/107, 86.9%), problem clarification and diagnostics (90/107, 84.1%), psychoeducation (80/107, 75.7%), and guiding and supporting self-management (65/107, 60.7%).

**Table 1:** Background information of the respondents and their hospital (N=107).

Characteristics		Statistics
**Gender, n (%)**		
	Men	15 (14.0)
	Women	92 (86.0)
Age (years), mean (SD)		40.5 (11.5)
**Professional background, n (%)**		
	Psychologist, MSc	13 (12.1)
	Health care psychologist	45 (42.1)
	Clinical psychologist	27 (25.2)
	Clinical neuropsychologist	2 (1.9)
	Other (eg, in training to become health care psychologist)	20 (18.7)
**Number of consultations during 1 complete treatment, n (%)**		
	<5	39 (36.4)
	5-10	60 (56.1)
	>10	8 (7.5)
Average number of minutes spent per therapy session, mean (SD)		53.9 (29.2)

### Internet Experience

Most respondents used the internet on a daily basis (102/107, 95.3%) and rated their internet skills as (very) good (92/107, 86%). Concerning work functionalities, respondents mainly used the internet to search for medical information (103/107, 96.3%) or have contact with clients through email (71/107, 66.4%). Regarding availability of Web-based health applications in the hospital, respondents primarily reported availability of electronic medical records (104/107, 97.2%), a website with patient information (73/107, 68.2%), and a Web portal with patient records (46/107, 43%). Electronic or Web-based screening (21/107, 19.6%), Web-based self-management modules (18/107, 16.8%), and use of teleconsultation (10/107, 9.3%) were less common.

### Use of Guided Internet-Based Cognitive Behavioral Therapy

Regarding the type of problems for which the respondents considered iCBT as an appropriate treatment, anxiety- and/or mood-related problems were most often reported (81/107, 75.7%), followed by problems dealing with chronic physical complaints and limitations (78/107, 72.9%), sleep problems (72/107, 67.3%), and fatigue problems (70/107, 65.4%).

Table 2 shows that 15.9% (17/107) of the respondents used guided iCBT at the time of filling in the survey. More than half of the respondents (68/107, 63.6%) had seen guided iCBT programs before. Among this subgroup, almost three-fourths (49/68, 72.1%) considered iCBT a suitable treatment for problems related to dealing with chronic physical complaints and limitations, and more than three-fourths (54/68, 79.4%) considered iCBT a suitable treatment for anxiety- and/or mood-related problems. In addition, almost one-third of the respondents (31/107, 29%) had received training in the use of guided iCBT and more than a quarter used it as a part of their work activities in the past (31/107, 29%). Within these subgroups, more than half of the respondents (18/31, 58.1% in both subgroups) considered iCBT a suitable treatment for problems related to dealing with chronic physical complaints and limitations, and more than three-fourths (26/31, 83.9% and 25/31, 80.6%, respectively) considered iCBT a suitable treatment for anxiety- and/or mood-related problems. Finally, of the (former) users, a majority (20/31, 64.5%) had used guided iCBT in less than 10 treatments. Regarding future use of iCBT, more than one-fourth of all nonusers (23/90, 25.6%) expected to use guided iCBT within the next year, and more than half (57/90, 63.3%) expected to use guided iCBT within 2 to 5 years.

### Facilitators and Barriers

Distribution of the scores on the UTAUT constructs was normal. Internal consistency was acceptable to excellent (see Table 3). On average, responses varied between the answer categories *neutral* and *partly agree*.

### Influence of Unified Theory of Acceptance and Use of Technology Constructs on Behavioral Intention Among Nonusers

Pearson correlations showed statistically significant moderate to high positive associations between the UTAUT constructs PE, EE, SI, and FC and the dependent variable of BI, respectively (PE *r*=0.656; EE *r*=0.479; SI *r*=0.251; and FC *r*=0.443; *P*<.001 for PE, EE, and FC, and *P*=.02 for SI). Table 4 shows the results of the multiple regression analysis using the enter method. The multiple regression model with the dependent variable BI explained 49% of variance in BI (*R*^2^=0.490; *F*_4.85_=20.405; *P*<.001). The constructs PE, EE, and FC had a significant positive effect on BI regarding use of iCBT, whereas SI did not.

**Table 2:** Use of guided internet-based cognitive behavioral therapy (N=107).

Question		Statistics, n (%)
Current use of guided iCBT^a^		17 (15.9)
**Previous experience with guided iCBT**		
	Has seen a program of guided iCBT	68 (63.6)
	Has received a training in the use of guided iCBT	31 (29.0)
	Has applied guided iCBT in previous treatments	31 (29.0)
**Completed guided iCBT treatment programs by (former) users (n=31)**		
	<10 treatments	20 (64.5)
	10-20 treatments	4 (12.9)
	>20 treatments	7 (22.6)
**Expected time frame of iCBT usage in the future among nonusers (n=90)**		
	Within the next year	23 (25.6)
	Within 2-5 years	57 (63.3)
	Not within the next 5 years	9 (10.0)
	Never	1 (1.1)

^a^iCBT: internet-based cognitive behavioral therapy.

**Table 3:** Scores on the Unified Theory of Acceptance and Use of Technology constructs (total N=107; users N=17; nonusers N=90).

Construct		Cronbach alpha	Statistics, mean (SD)^a^
**Behavioral intention**		.90	3.9 (.8)
	Users	—^b^	4.5 (.7)
	Nonusers	—	3.8 (.8)
**Performance expectancy**		.80	3.7 (.4)
	Users	—	3.7 (.3)
	Nonusers	—	3.7 (.4)
**Effort expectancy**		.71	3.5 (.4)
	Users	—	3.5 (.3)
	Nonusers	—	3.5 (.4)
**Social influence**		.78	3.0 (.7)
	Users	—	3.2 (.7)
	Nonusers	—	2.9 (.6)
**Facilitating conditions**		.68	3.4 (.6)
	Users	—	3.5 (.6)
	Nonusers	—	3.3 (.6)

^a^Responses answered all statements of all constructs by a 5-point Likert scale, namely 1: completely disagree, 2: partly disagree, 3: neutral, 4: partly agree, and 5: completely agree.

^b^Not applicable.

**Table 4:** Results multiple regression analysis of Unified Theory of Acceptance and Use of Technology constructs on behavioral intention (N=90).

Model	B^a^	Beta^b^	SE	*P* value
Constant	−1.823	—^c^	0.669	.01
Performance expectancy	0.803	0.438	0.176	<.001
Effort expectancy	0.625	0.288	0.187	.001
Social influence	−0.222	−0.174	0.139	.11
Facilitating conditions	0.355	0.270	0.154	.02

^a^B: partial regression coefficient.

^b^Beta: standardized regression coefficient.

^c^Not applicable.

## Discussion

### Principal Findings

Guided iCBT is a suitable and effective treatment for patients with a chronic somatic illness. However, its implementation in clinical practice remains challenging. The main purpose of this study was to examine the fit and the current degree of implementation of iCBT among psychologists in medical settings and determine its facilitators and barriers among nonusers. Most respondents in our sample were female (92/107, 86.0%) and had an official registration as psychologist, which is representative for the Dutch situation [30].

The results of the study showed that the use of technology is highly integrated among psychologists, both on a personal level and work-related level. Moreover, respondents generally considered iCBT as an appropriate treatment for the type of problems they treat their patients for, regardless of their (previous) experience with iCBT. The actual current use of iCBT, however, was limited. Our results show that only 16% (17/107) of the respondents is currently using iCBT. In addition, 29% (31/107) had received training in the use of guided iCBT, and 29% (31/107) had used it as a part of their work activities in the past. This raises the question why only such a small portion of psychologists actually use these Web-based treatment tools nowadays and what happened when they stopped using them. Presumable reasons for ending the use of iCBT could be that people have changed organizations and were not satisfied with the Web-based programs they used or, for instance, a change of policy from their management.

Our results concerning outcomes on the UTAUT model provide some insight into these questions and into what is further needed to increase implementation success. BI regarding the use of iCBT was found to be high among nonusers, with 98.9% (89/90) planning to use it within the upcoming 5 years; therefore, there is a high willingness to use Web-based tools in treatment. We also found that PE, EE, and FC appear to be significant predictors for this BI. These results indicate that aspects such as effectiveness for the client population, easiness of use, and managerial focus impact psychologists’ choice in the use of iCBT. These outcomes are in line with recent research on iCBT use in primary and routine care [26,27]. Still, the answers on most constructs had a small dispersion (spreading of the answers; responses were located around the answer categories *partly agree* or *neutral*), indicating that the respondents did not have very outspoken expectations on these determinants. This might indicate an implementation barrier in itself because the needs and beliefs about iCBT might not be strong enough to actually create behavioral change. To create actual change, it seems of major importance that the availability of Web-based self-management tools increases in hospital settings because, currently, only 17% (18/107) have access to iCBT. As a result, a larger part of psychologists in these settings will actually be able to use iCBT when willing to.

In the Netherlands, there is quite a large number of commercially provided iCBT portals, in which hosting and updating is outsourced. Psychologists are able to make use of these programs using a paid license. Although we now know that practicing psychologists would be intended to use these iCBT services, the actual access is probably often depending on the willingness of their management to invest in iCBT services. Future implementation efforts should therefore initially focus on these types of stakeholders, using implementation models that take a broad range of stakeholders into account. An example of such a model is the Consolidated Framework for Implementation Research [31], addressing 5 domains to include in implementation research. These are intervention characteristics (eg, adaptability and relative advantage), the setting within the organization (eg, culture, leadership engagement, and communication), the environment outside the organization (eg, external policies and incentives, peer pressure, needs, and resources), characteristics of the individuals involved (eg, self-efficacy, personal attributes, and individual stages of change), and the process of implementation (eg, planning, execution, and internal implementation leaders).

### Future Directions

Based on our results using the UTAUT model, we can further reflect on what follow-up steps in improving implementation success seem essential, partly on an individual level. Concerning PE and EE, this study showed that fewer than one-third of the respondents have received a training in the use of iCBT. Therefore, expectations about whether iCBT asks a lot of new skills, affects work productivity, and increases the quality of care provision appear to affect the implementation process of iCBT. Moreover, these findings are consistent with previous research stating that lack of knowledge of the program and expectations regarding effectiveness and fully mastering the required protocol influence acceptance of iCBT [8,27,32,33]. Therefore, attention should be paid to providing employees more education in iCBT by offering knowledge with regard to availability of existing iCBT programs, their effectiveness, usefulness, and performance productivity and by offering them proper skills training [27,34]. Regarding FC, stimulation of iCBT use from a management level is a key prerequisite for use in daily practice. Moreover, attention should be paid to the degree of customization of iCBT to the daily workflow of psychologists.

### Limitations

With this survey, we aimed to reach the most representative sample possible by trying to contact each psychologist in a medical setting in the Netherlands. The possibility exists that people interested in electronic health (eHealth) or actual eHealth users were more inclined to respond to the survey. This could result in fewer psychologists actually using iCBT in the complete population than the results from this survey show. In addition, social desirability may have influenced the provided answers of the respondents because the government encourages the use of eHealth among health professionals, which might have pressured our respondents toward positive answers. Furthermore, it is not possible to exactly estimate how many psychologists actually received our survey because invited psychologists were asked to forward our invitation to colleagues to whom it might be relevant as well. However, our estimated response rate of 24.8% is in line with the average response rate of large surveys [35]. Moreover, by sending out 2 reminders each 7 days after the first invitation email and by making use of personalization, appropriate measures were used to increase the response rate as much as possible [36]. Delay between reminders was based on research stating no significant difference in response rate for follow-up emails sent after 1 or 2 weeks and recommending time lags of 1 week [37].

### Conclusions

Despite an average to high BI, current implementation of iCBT is low among psychologists in medical settings. Increasing the availability of iCBT and bringing change in other areas that could influence the gap between intention and actual use is needed. The UTAUT constructs PE, EE, and FC significantly affect BI regarding use of iCBT. Therefore, further research and implementation trajectories should focus on offering a wide range of iCBT programs, education on iCBT facilities and required skills, and customization of iCBT to the daily workflow of psychologists.

## Appendix

Multimedia Appendix 1 English version of the survey as distributed among medical psychologists in the Netherlands.

## Abbreviations

BI: behavioral intention
CBT: cognitive behavioral therapy
eHealth: electronic health
EE: effort expectancy
FC: facilitating conditions
iCBT: internet-based cognitive behavioral therapy
MHCs: mental health counselors
PCPs: primary care psychologists
PE: performance expectancy
SI: social influence
UTAUT: Unified Theory of Acceptance and Use of Technology
