# Supplementary material for: Internet-Based Cognitive Behavioral Therapy Among Psychologists in a Medical Setting: A Survey on Implementation
Source: J Med Internet Res. 2019 Aug 9;21(8):e13432. doi: 10.2196/13432 (PMC6713041; doi:10.2196/13432)
Supplement: Multimedia Appendix 1 [file jmir_v21i8e13432_app1.pdf]

Multimedia Appendix 1. English version of the survey as distributed among medical psychologists in the Netherlands

You and your practice

1	What is your age?	……… years
2	What is your sex?	☐ Male
		☐ Female
3	What is your professional background?	☐ Psychologist, MSc.
		☐ Healthcare psychologist
		☐ Clinical psychologist
		☐ Clinical neuropsychologist
		☐ Other ………
4	Are you a member of the National Association of Medical Psychology?	☐ Yes
		☐ No
5	How many years do you work as a psychologist?	……… years
6	How many years do you work as a *medical* psychologist?	……… years
7	How many hours are you employed on a weekly basis?	……… hours a week
8	In which province is your hospital positioned?	☐ Groningen
		☐ Friesland
		☐ Drenthe
		☐ Overijssel
		☐ Gelderland
		☐ Utrecht
		☐ Flevoland
		☐ Noord-Holland
		☐ Zuid-Holland
		☐ Noord-Brabant
		☐ Zeeland
		☐ Limburg
9	What kind of hospital do you work for?	☐ General hospital
		☐ Top clinical hospital
		☐ Academic hospital
		☐ Specialized hospital
10	How many inhabitants does the place in which the hospital is located count?	☐ < 10.000 inhabitants
		☐ 10.000 – 50.000 inhabitants
		☐ 50.000 – 150.000 inhabitants
		☐ 150.000 – 300.000 inhabitants
		☐ > 300.000 inhabitants
11	How many medical psychologists work at your hospital?	…….. medical psychologists
12	With what reason/referral indication do clients mostly come to you as a medical psychologist? (max 3 answers)	☐ Problems in dealing with the chronic psychical complaints and limitations
		☐ Anxiety and/or mood complaints
		☐ Social problems (e.g. relationships)
		☐ Pain problems
		☐ Sleep problems
		☐ Fatigue complaints
		☐ Sexuality problems
		☐ Others, namely ………
13	How many new clients do you see every month, on average?	……… clients

14	Per treatment program, how many sessions do you have with a client, on average?	☐ 1 session
		☐ 2 to 5 sessions
		☐ 5 to 10 sessions
		☐ More than 10 sessions
15	How many minutes do you spend per client contact, on average?	……… minutes
16	What type of help do you mainly offer your clients? (max 3 answers)	☐ Problem clarification and diagnostics
		☐ Psycho-education
		☐ Guidance/support with self-management
		☐ Interventions aimed at improving physical functioning of the client
		☐ Interventions aimed at improving psychological functioning of the client
		☐ Relapse prevention
		☐ Other, namely ………

You, your hospital, and technology

17	How often do you use the internet? (in general, i.e. to send an e-mail, or to search for information)	☐ (Almost) every day
		☐ Several times a week
		☐ Approximately 1 day a week
		☐ Less than 1 day a week
		☐ (Almost) never
18	How do you rate your internet skills?	☐ Very good
		☐ Good
		☐ Average
		☐ Poor
		☐ Very poor
19	Do you also use the internet for work?<br>If so, please indicate the purpose.<br>(you can select multiple answers)	☐ No, never
		☐ Yes, to search for medical information
		☐ Yes, to search for information on insurances and reimbursements
		☐ Yes, to communicate with clients via e-mail
		☐ Other purpose, namely …….
20	Which technology is available in your hospital?<br>(you can select multiple answers)	☐ Electronic medical records
		☐ Web portal with client file
		☐ Online appointment tool
		☐ Online recipes tool
		☐ Website for client information
		☐ Electronic/online screening
		☐ eConsult (secure client e-mail contact)
		☐ Tele-medicine (e.g. video calling, remote care)
		☐ Online self-management/treatment modules
		☐ None of the above-mentioned possibilities
		☐ Other, namely ………

<u>You, your hospital, and guided eHealth programs</u>

Within hospital setting, eHealth programs can be offered in the form of guided internet-based cognitive behavioural therapy (guided-iCBT), which makes it suitable for self-management support for patients with chronic somatic complaints, and treatment of co-morbid anxiety and mood problems.

21	Have you ever seen a program for guided eHealth?	☐ Yes
		☐ No
		☐ I don't know
22	Have you ever been trained to use a program for guided eHealth?	☐ Yes
		☐ No
		☐ I don't know
23a	Have you ever used a program for guided eHealth within treatment?	☐ Yes
		☐ No
		☐ I don't know
23b	If so, how many guided eHealth treatment programs did you go through, approximately?	☐ Zero treatments / I don't know
		☐ Less than 10 treatments
		☐ Between 10 and 20 treatments
		☐ More than 20 treatments
24	For which type of complaints do you think guided eHealth programs are suitable/did you use guided eHealth programs?<br>(you can select multiple answers)	☐ Problems in dealing with the chronic psychical complaints and limitations
		☐ Anxiety and/or mood complaints
		☐ Social problems (e.g. relationships)
		☐ Pain problems
		☐ Sleep problems
		☐ Fatigue complaints
		☐ Sexuality problems
		☐ Others, namely ………

25. I expect/perceive that guided eHealth programs…*

			Totally disagree	Partly disagree	Neutral	Partly agree	Totally agree
a	[PE]	… be effective for my client population.	☐	☐	☐	☐	☐
b	[EE]	… be easy to use.	☐	☐	☐	☐	☐
c	[PE]	… be useful for my client population.	☐	☐	☐	☐	☐
d	[PE]	… increase the quality of my care provision.	☐	☐	☐	☐	☐
e	[SI]	… advised by important people or colleagues in my immediate area.	☐	☐	☐	☐	☐
f	[EE]	… ask a lot of practice and schooling.	☐	☐	☐	☐	☐
g	[PE]	… increase the diversity of my care provision.	☐	☐	☐	☐	☐
h	[FC]	… fit within the financing structures of my practice.	☐	☐	☐	☐	☐
i	[EE]	… are time consuming to use.	☐	☐	☐	☐	☐
j	[FC]	… fit within the current technological state of things in my practice.	☐	☐	☐	☐	☐
k	[PE]	… increase the productivity of my practice.	☐	☐	☐	☐	☐
l	[SI]	… be seen as a positive development among my colleagues.	☐	☐	☐	☐	☐
m	[PE]	… increase the interaction or communication with my clients.	☐	☐	☐	☐	☐
n	[FC]	… be stimulated by my organization or manager.	☐	☐	☐	☐	☐
o	[EE]	… be fun to use.	☐	☐	☐	☐	☐
p	[SI]	… used by colleagues with a lot of respect in my hospital.	☐	☐	☐	☐	☐
q	[PE]	… decrease the interaction or communication with my clients.	☐	☐	☐	☐	☐
r	[PE]	… increase the tailoring of care among my clients.	☐	☐	☐	☐	☐
s	[EE]	… ask a lot of new skills from me.	☐	☐	☐	☐	☐
t	[EE]	… are easy to gain skills in.	☐	☐	☐	☐	☐
u	[SI]	… be actively used by my colleagues.	☐	☐	☐	☐	☐
v	[EE]	… be interesting to use.	☐	☐	☐	☐	☐
w	[FC]	… fit within my way of working.	☐	☐	☐	☐	☐
x	[SI]	… be seen as a positive development by my organization or manager.	☐	☐	☐	☐	☐
y	[FC]	… fit with the goals of (the management of) my organization.	☐	☐	☐	☐	☐
z	[SI]	… cause an increase in respect and admiration to colleagues who use this.	☐	☐	☐	☐	☐
aa	[FC]	… fit with the possibilities I receive for schooling and education.	☐	☐	☐	☐	☐

* [PE] = Performance Expectancy
[EE] = Effort Expectancy
[SI] = Social Influence
[FC] = Facilitating Conditions

			Totally disagree	Partly disagree	Neutral	Partly agree	Totally agree
26	[BI]	I would like to use/keep using guided eHealth programs.	☐	☐	☐	☐	☐
27	[BI]	I intend to use/keep using guided eHealth programs.	☐	☐	☐	☐	☐
28	[BI]	I expect to use/keep using guided eHealth programs in the future.	☐	☐	☐	☐	☐
29		It is/remains my own choice to continue using guided eHealth programs.	☐	☐	☐	☐	☐
30		My management will put pressure on me to continue using guided eHealth programs.	☐	☐	☐	☐	☐

31	[BI]	Within which time frame do you expect to use guided eHealth programs?	☐ I currently use them already
			☐ Within the next six months
			☐ Within the next year
			☐ Within two to five years
			☐ Not within the next five years
			☐ Never
32		Are you interested in receiving (more) information about guided eHealth programs?	☐ Yes
			☐ No
33		Are you interested in a training course in the use guided eHealth programs?	☐ Yes
			☐ No
34		If so, what kind of information or training would you like to receive?	....

* [BI] = Behavioral Intention
